# Exploiting Polyelectrolyte Complexation for the Development of Adhesive and Bioactive Membranes Envisaging Guided Tissue Regeneration

**DOI:** 10.3390/jfb14010003

**Published:** 2022-12-20

**Authors:** Mário C. Fonseca, Ana Catarina Vale, Rui R. Costa, Rui L. Reis, Natália M. Alves

**Affiliations:** 1 3B’s Research Group, I3Bs—Research Institute on Biomaterials, Biodegradables and Biomimetics, University of Minho, Headquarters of the European Institute of Excellence on Tissue Engineering and Regenerative Medicine, AvePark, Parque de Ciência e Tecnologia, Zona Industrial da Gandra, Barco, 4805-017 Guimarães, Portugal; 2 ICVS/3B’s—PT Government Associate Laboratory, 4710-057 Braga, Portugal

**Keywords:** polyelectrolyte complexation, chitosan, hyaluronic acid, catechol, bioactive glass nanoparticles

## Abstract

Mussels secrete protein-based byssal threads to tether to rocks, ships, and other organisms underwater. The secreted marine mussel adhesive proteins (MAPs) contain the peculiar amino acid L-3,4-dihydroxyphenylalanine (DOPA), whose catechol group content contributes greatly to their outstanding adhesive properties. Inspired by such mussel bioadhesion, we demonstrate that catechol-modified polysaccharides can be used to obtain adhesive membranes using the compaction of polyelectrolyte complexes (CoPEC) method. It is a simple and versatile approach that uses polyelectrolyte complexes as building blocks that coalesce and dry as membrane constructs simply as a result of sedimentation and mild temperature. We used two natural and biocompatible polymers: chitosan (CHI) as a polycation and hyaluronic acid (HA) as a polyanion. The CoPEC technique also allowed the entrapment of ternary bioactive glass nanoparticles to stimulate mineralization. Moreover, combinations of these polymers modified with catechol groups were made to enhance the adhesive properties of the assembled membranes. Extensive physico-chemical characterization was performed to investigate the successful production of composite CoPEC membranes in terms of surface morphology, wettability, stability, mechanical performance, in vitro bioactivity, and cellular behavior. Considering the promising properties exhibited by the obtained membranes, new adhesives suitable for the regeneration of hard tissues can be envisaged.

## 1. Introduction

Membranes are remarkable biomedical devices that can be endowed with physico-chemical properties to aid in wound healing, permselectivity for hemodialysis, and controlled cell culture and expansion [1,2,3]. Novel advances have focused on the development of composite bioabsorbable membranes with enhanced bioactivity, with an emphasis on compositions made exclusively of polymeric biomaterials. In these formulations, the polymer acts as a matrix for cell growth, which is conveniently supported by intrinsic properties such as biodegradability, biocompatibility, and flexibility. In contrast, inorganic compounds, such as glass nanoparticles, can improve mechanical properties and provide bioactivity to the device (e.g., osteoconductivity in the glass nanoparticles example) [4,5]. Improved mechanical properties and mineralization have found a growing interest for guided bone regeneration (GBR) since these biomaterials specialize in promoting self-reparation in bone and in surrounding damaged tissues, such as in periodontal disease [6]. The osteoconductivity of the ceramics combined with the multifunctionality of the polymers could then create mimics of the native bone extracellular matrix (ECM). Therefore, composites based on biodegradable polymers and bioactive ceramics or glasses can be more effective than each material alone in regenerating the structure and function of hard tissues.

The layer-by-layer technique (LbL) and its variants (e.g., spin-, spray-, and dip-coating) have been used to assemble hybrid coatings and films for tissue regeneration due to their sole reliance on the spontaneous attraction between charged species [7]. In this method, layers of polyelectrolytes deposit alternately on top of oppositely charged surfaces. The fact that electrostatic interactions are non-covalent bonds enables natural polyelectrolytes to be used as building blocks, including charged materials from the ECM. As such, LbL has promoted the assembly of devices with high cellular viability, capacity for targeted drug delivery, and biomineralization [8]. However, the main drawback of LbL is the long deposition time of each deposition cycle, which can last several minutes, thus prolonging the membrane production time, especially when a large number of layers is desired. We hypothesized that the assembly of polyelectrolyte-based membranes could be significantly accelerated using a one-pot quasi-LbL approach. The methodology of compaction of polyelectrolyte complexes (CoPEC) is a suitable tool to achieve this end; it can use the traditional charged species of LbL, but instead of sequential deposition stages, the polyelectrolytes are mixed beforehand and a membrane is formed simply by the sedimentation of the complexes [9]. Membranes of this class have already been shown to be promising candidates for soft tissue substitutes [10,11]. However, due to the preference in using polymeric materials, they have not yet been studied for hard tissue regeneration.

In this study, we employed the CoPEC methodology to produce adhesive and osteoconductive membranes. We chose chitosan (CHI) and hyaluronic acid (HA) as polymeric constituents of the membranes since they are polysaccharides that resemble many native ECM biomolecules [12,13]. To ensure high adhesiveness, inspiration was drawn from the mussels’ adhesive proteins (MAPs), which can establish strong bonds between mussels and different structures, including in wet conditions [14,15,16]. The outstanding adhesive properties of MAPs are ascribed mainly to the amino acid 3,4-dihydroxy-L-phenylalanine (DOPA), its analog dopamine, and the catechol groups exhibited by this amino acid. Previous work by our group [17,18] has used bioactive glass nanoparticles (BGNPs) using LbL approaches and demonstrated great potential for mineralizing into a crystalline hydroxyapatite interface. As such, they make excellent ceramic candidates for quasi-LbL methods as well.

In order to investigate the suitability of the CoPEC method to incorporate such a wide number of biomaterial classes, different combinations of both biopolymers—modified or not with catechol groups—and BGNPs were tested. These combinations were expected to exhibit strong adhesive properties and favorable mineralization and cellular behaviors. An extensive physico-chemical characterization was conducted with the different CoPEC membranes, focusing on their surface morphology, wettability, stability, mechanical performance, in vitro mineralization, and cellular behavior. Ultimately, we adjudged the best CoPEC membrane condition for guided regeneration of bone defects.

## 2. Materials and Methods

### 2.1. Materials

Medium molecular weight CHI (190–300 kDa) with a degree of deacetylation (DD) between 75% and 85% was purchased from Sigma-Aldrich (St. Louis, MO, USA). Prior to use, CHI was deacetylated and purified by a recrystallization process. Sodium hyaluronate was purchased from Lifecore (Chaska, MN, USA), with an average molecular weight of 3.27 × 10^5^ Daltons. Dopamine (DN) hydrochloride and hydrocaffeic acid (HCA), magnesium chloride hexahydrate (MgCl_2_·6H_2_O), sodium sulphate (Na_2_SO_4_), 2-(4-(2hydroxyethyl)-1-piperazinyl) ethane sulfonic acid (HEPES, C_8_H_18_N_2_O_4_S), hydrochloric acid (HCl), tetraethyl orthosilicate (TEOS, 99.90% pure), citric acid monohydrate (99%), ammonium phosphate dibasic, calcium nitrate tetrahydrate (99%), ethanol absolute, and ammonia water (ammonium hydrogen phosphate (98%), maximum of 33% NH_3_) were purchased from Sigma-Aldrich (St. Louis, MO, USA). Sodium hydroxide (NaOH) was purchased from Fisher Chemical (Fisher Scientific UK, Leics, UK). Sodium hydrogen carbonate (NaHCO_3_), dipotassium hydrogen phosphate trihydrate (K_2_HPO_4_·3H_2_O), potassium chloride (KCl), and calcium chloride (CaCl_2_) were purchased from Merck KGaA (Darmstadt, Germany). For cellular assays, complete Dulbecco’s modified minimum essential medium with low glucose and phenol red, sodium bicarbonate, and fetal bovine serum were purchased from Sigma-Aldrich (St. Louis, MO, USA). Antibiotic/antimycotic and TrypLETM express solution with phenol red was acquired from Life Technologies (Paisley, UK), and CellTiter 96^®^ AQueous One Solution Cell Proliferation Assay was purchased from Promega (Madison, WI, USA).

### 2.2. Synthesis of the Chitosan–Catechol Conjugate (CHI–Cat)

The synthesis of the chitosan–catechol conjugate was performed following procedures reported by Moreira et al. [18] with some modifications based on several previous studies [19,20,21]. CHI modification with catechol groups was accomplished by 1-ethyl 3 (3 dimethylaminopropyl)carbodiimide hydrochloride (EDC), which is a zero-length activation agent used to couple carboxyl groups to primary amines. First, 100 mL of 1% (*w*/*v*) chitosan solution in a 1% hydrochloric acid (HCl) solution (5.5 M) were prepared. Under protection of aluminum foil, 5 mL of a 3,4-dihydroxyhydrocinnamic acid solution, also known as hydrocaffeic acid (HCA) (59 mg.mL^−1^), was prepared in osmotized water, and 6 mL of EDC solution (119 mg.mL^−1^) was prepared in a mixture of osmotized water and ethanol (1:1, *v*/*v*). Next, the EDC solution was added to the CHI solution, which was almost immediately followed by the addition of the HCA solution. The solution was kept under stirring conditions at room temperature. To obtain a final pH of 4.8, the necessary amount of 1 M NaOH solution was added. The reaction was maintained for 18 h under a nitrogen atmosphere protected from light. The product was dialyzed against an acidic aqueous solution (pH 5.0, HCl solution) for three days at 4 °C, followed by only osmotized water for 4 h using a membrane with molecular weight cut-off of 3.5 kDa. The product was freeze-dried for 4 days and then stored at −20 °C.

### 2.3. Synthesis of the Hyaluronic Acid–Catechol Conjugate (HA–Cat)

The synthesis of the HA–cat conjugate was performed based on the procedure described by Moreira et al. [18], which was adapted from a previous study [22]. This procedure also relied on synthesis using carbodiimide chemistry. Briefly, HA (1 g) was dissolved in 100 mL of PBS and the pH was adjusted to 5.5 using 0.5 M HCl or 0.5 M NaOH aqueous solutions. The solution was purged with nitrogen for at least 1 h to limit oxygen interaction with the solution. At a temperature of 4 °C and in a dark room, 338 mg of EDC and 474 mg of dopamine were added to the previous HA solution and maintained under slow stirring for 2 h or until the complete dissolution of the reagents. The unreacted chemicals and urea byproducts were removed by dialysis against osmotized water at 4 °C for one week. The product was lyophilized for four days and, after that, stored in the dark at −20 °C.

### 2.4. Production of the Ternary Bioactive Glass Nanoparticles (BGNPs)

The production of the ternary bioactive glass nanoparticles was performed through a sol-gel method described by Almeida et al. [17] with some modifications in the washing step in order to obtain ternary bioactive glass nanoparticles with SiO_2_:CaO:P_2_O_5_ (mol%) = 55:40:5. Briefly, calcium nitrate (7.639 g) was dissolved in 120 mL of osmotized water at room temperature. TEOS (9.8353 mL) was then diluted in 60 mL of ethanol and added to the calcium nitrate solution. The pH was adjusted to 2 with 30 mL of citric acid solution (10%) and left under stirring conditions for 3 h, resulting in solution A. Solution B was prepared by dissolving 1.078 g of ammonium phosphate dibasic (NH_4_)_2_HPO_4_ in 1500 mL of osmotized water, and the pH was adjusted to 11.5 with an ammonium hydroxide (NH_4_OH) solution. Solution A was added to solution B drop-wise under vigorous stirring controlling the pH at 11.5 with the NH_4_OH solution. The resulting solution was stirred for 48 h and then left aging for 24 h to promote particle precipitation. The solution was washed 20 times with 500 mL of osmotized water. The obtained gel was freeze-dried for seven days and finally calcinated (100 °C per hour) at 700 °C for 3 h to obtain a white gel powder.

### 2.5. Ultraviolet-Visible Spectrophotometry Characterization

A Shimadzu UV-1601 spectrometer was used for the ultraviolet-visible spectrophotometry analysis to determine the DD of CHI. First, several solutions of N-acetylglucosamine were dissolved in HCl at various concentrations to obtain a calibration curve. To calculate the DD, the absorbance was measured at 199 nm. The spectra obtained between 200–350 nm were used to confirm the success of conjugating CHI and HA with catechol functional groups. Quantification of the degree of catechol substitution was made via a colorimetric assay and analysis of the absorbance maximum at around 280 nm. HCA and DN solutions were prepared to create a catechol concentration standard curve and quantify the catechol content of CHI–cat and HA–cat, respectively.

### 2.6. FTIR Spectroscopy Analysis

Fourier transform infrared spectroscopy (FTIR, Shimadzu IRPrestige-21, Kyoto, Japan) was used to confirm the presence of the main functional groups of the polymers and the BGNPs, operating under attenuated total reflectance (ATR) mode. The FTIR spectra were recorded from 600 to 4000 cm^−1^ in transmittance mode at a resolution of 4 cm^−1^.

### 2.7. Zeta Potential (ζ) Measurements

To determine the order of mixing of the polyelectrolytes and the stoichiometry of the complexation, the polycations (CHI, CHI–cat) and the polyanions (HA, HA–cat) prepared in the same conditions as for the CoPEC methodology were titrated. Typically, 100 μL of one polyelectrolyte was added stepwise to a fixed volume of 1 mL of the other polyelectrolyte, and the ζ-potential of the complexes was measured with each titration using a ZEN3600 nano-sizer (Malvern, UK) until the ζ-potential of the complexes reached ≈0 mV. The charge of the BGNPs was also characterized by this method. Suspensions of 0.025 mg.mL^−1^ of BGNPs were prepared in PBS (pH = 7.4) and sonicated for 15 min before the measurements to avoid their agglomeration. These measurements were performed in triplicate at 25 °C.

### 2.8. Production of Membranes through CoPEC Methodology

**Figure 1:**
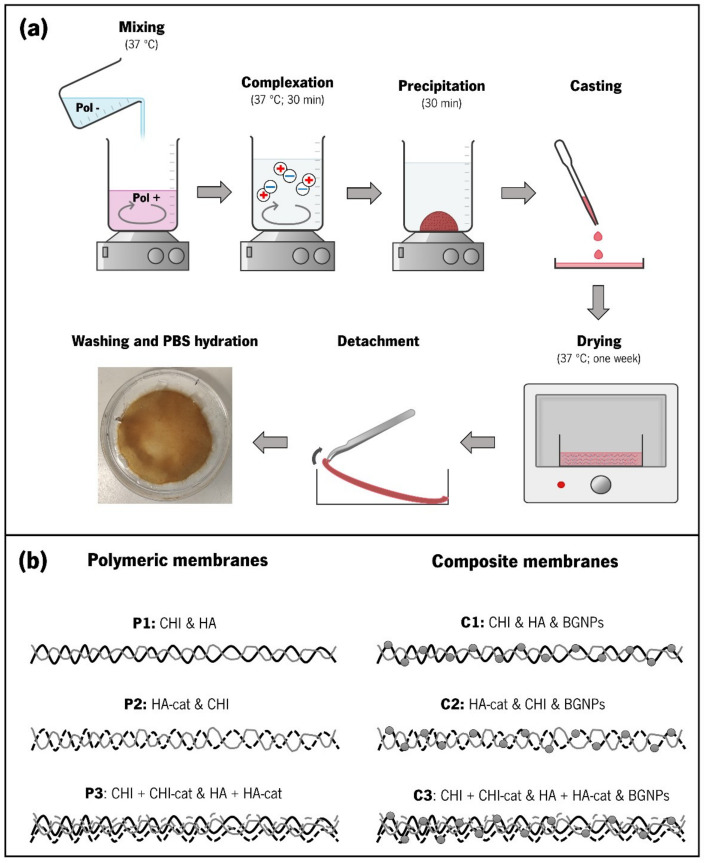
(**a**) Scheme illustrating the various steps involved in the employed CoPEC membrane production technique. The micrograph under “washing and PBS hydration” shows a membrane assembled from CHI–cat and HA–cat in a Petri dish (diameter = 5 cm); (**b**) Composition of the different membranes fabricated by CoPEC. The nomenclatures attributed to each condition—P1, P2, P3, C1, C2, and C3—are indicated.

The production of the membranes was adapted from the sedimentation/evaporation protocols described in previous studies (Figure 1) [10,11]. For the polymeric membranes, the CoPEC process starts with the preparation of two solutions with a concentration of 2.5 mg.mL^−1^ (one containing the polycation and another containing the polyanion) using 0.15 M sodium chloride (NaCl) as the solvent. When using unmodified CHI, 0.15 M NaCl in 1.5 % (*v*/*v*) acetic acid was used since it is only soluble in acidic conditions. When using BGNPs, the nanoparticles were mixed in the polycation solution and then sonicated for 1 min.

Each solution’s pH was adjusted to 5.5 using 1 M HCl or 0.5 M NaOH and heated to 37 °C. Next, the polyanion solution was slowly poured into the polycation solution under stirring, and they continued stirring for 30 min to complete the complexation of the polyelectrolytes. After that, the resulting PECs were left to precipitate for another 30 min. After manually removing the supernatant, the remaining suspension was evenly distributed in round polystyrene Petri dishes and dried at 37 °C for seven days. The dry membranes were then stored at room temperature. The pure polymeric and the composite membrane compositions produced through the CoPEC method are illustrated in Figure 1a.

### 2.9. Morphological and Topographic Characterization of the CoPEC Membranes

Membrane surface morphology, cross-sections, and element compositions were studied using a scanning electron microscope (SEM) JSM-6010 LV (JEOL, Tokyo, Japan) coupled with EDS (INCAx-Act, PentaFET Precision, Oxford Instruments, Abingdon, UK). For analysis of the cross-section, the membranes were fractured after being immersed in nitrogen. With the exception of EDS analysis, the samples were sputtered with a thin gold layer using an EM ACE600 sputter coater (Leica Microsystems, Wetzlar, Germany) prior to observation. The dry thickness of the CoPEC membranes was measured with the assistance of ImageJ software (US National Institutes of Health, Bethesda, MD, USA, version 1.53 k).

### 2.10. Wettability and Surface Energy Analysis

Water contact angles (WCA) of the upper side of the CoPEC membranes were assessed by the sessile drop method using water in an OCA15 Plus goniometer (DataPhysics Instruments, Filderstadt, Germany). Ten drops of 3 µL were dispensed with the aid of a motor-driven syringe, and the angle was measured immediately at room temperature. The surface energy (SE) of the membranes was also determined by the sessile drop method using water and diiodomethane and was calculated with the Owens, Wendt, Rabel, and Kaeble (OWRK) method. The SE calculations considered a water surface energy of 72.80 mN.m^−1^ and of 50 mN.m^−1^ for diiodomethane.

### 2.11. Water Uptake (WU) and Weight Loss (WL)

The CoPEC membranes were first weighed in the dry state and then soaked in PBS at 37 °C. The wet weight was measured after soaking the specimens for 15 min, 30 min, 1 h, 2 h, 3 h, 24 h, and 48 h. At each time point, the membranes were removed from the PBS solution, and the excess of PBS was removed with filter paper. The *WU* was calculated using Equation (1),

(1) $$WU=\frac{W_{w}-W_{d}}{W_{d}}\times 100$$

where 𝑊_𝑤_ is the weight of the hydrated membranes and 𝑊_𝑑_ is the initial weight in dry conditions. For each condition, three samples were tested, and the average value was defined as the *WU*.

For degradation studies, the CoPEC membranes were first weighed in the dry state and then were soaked either in a control PBS solution or PBS containing lysozyme from chicken egg white (0.013 mg.mL^−1^) for 31 days at 37 °C under agitation. After that, the membranes were retrieved, washed multiple times with ultra-pure Milli-Q^®^ water, dried, and then weighed. The weight loss was calculated using Equation (2),

(2) $$WL\;\left(\%\right)=\frac{W_{i}-W_{f}}{\;W_{i}}\times 100$$

where 𝑊_i_ is the initial weight of the dry sample and 𝑊_𝑓_ is the final weight of the dry sample after soaking for 31 days.

### 2.12. Mechanical Characterization

Tensile tests were carried out on a universal electromechanical testing machine (Instron 5543, Norwood, MA, USA) equipped with a tensile-capable 50 N load cell. All samples were pre-soaked in PBS and cut as specimens 10 mm wide and 30 mm long. The thickness was measured three times (*n* = 3) with a digital micrometer (Mitutoyo, Kawazaki, Japan), which was necessary to calculate the area of the cross-section in wet conditions. The measurements were made at a loading speed of 10 mm.min^−1^. The tensile strength at break (MPa), the Young’s moduli (MPa), and the tensile strain at break (ε, %) were determined from the obtained stress-strain curves.

The adhesiveness of the membranes was measured by performing lap-shear in accordance with the ASTM D1002 standard using the same load cell, displacement speed, and specimen dimensions as in the tensile tests. Membranes were pre-soaked in PBS, placed in contact with an overlapping area of 10 × 7 mm^2^, low-pressed between two glass slides, and hydrated in PBS solution overnight at 37 °C. The obtained stress-strain curves were used to calculate both the tensile stress (σ, kPa) and tensile strain (ε, %) at break.

Dynamic mechanical analysis (DMA) experiments were performed using TRITEC 2000B DMA equipment from Triton Technology, Keyworth, Nottinghamshire, UK). The samples were cut into 5 × 15 cm^2^ specimens, and the thickness of each sample was measured using a digital micrometer (Mitutoyo, Kawazaki, Japan). The samples were pre-soaked in a PBS solution, and the membranes were analyzed in a PBS bath at 37 °C. The membranes were clamped in the DMA apparatus at a 5 mm grip distance. The DMA spectra were obtained from five specimens of each membrane type subjected to a frequency scan between 0.1 and 10 Hz.

### 2.13. In Vitro Bioactivity Studies

Standard in vitro bioactivity studies were conducted by immersing three samples of each membrane composition in a freshly prepared simulated body fluid solution (1.0 SBF) [23] at 37 °C for 31 days. After that time, the samples were carefully washed in ultra-pure Milli-Q^®^ water and dried at room temperature for 24 h. The elemental composition of the apatite layer formed on their surface was studied by SEM coupled with energy dispersive X ray spectroscopy (JEOL, Tokyo, Japan), by Fourier transform infrared spectroscopy (Infrared spectrometer-Jasco) in the attenuated total reflectance (ATR) transmission mode (spectra found in Figure S1 of the Supplementary Materials), and by X-ray diffraction (XRD) using a Bruker AXS D8 Discover model (Bruker, Kontich, Belgium) operated at 40 kV and 40 mA using Cu Kα radiation. The XRD detector was scanned over a range of 2θ angles from 15° to 60° at a step size of 0.04° and dwell time of 1 s per step. The analysis for phase identification was performed using the analytical software EVA. The crystalline phases were identified and compared to the ICDD-2015 database (International Center for Diffraction Data).

### 2.14. Cellular Assays

SaOs-2 osteoblast-like cells from human osteosarcoma were obtained from the European Collection of Cell Cultures (ECA CC, Salisbury, UK). Cells were routinely cultured in 150 cm^2^ tissue culture flasks in complete Dulbecco’s modified minimum essential medium (DMEM) with low glucose and phenol red, supplemented with 3.7 g.dm^−3^ of sodium bicarbonate, 10% fetal bovine serum (FBS), and 1% penicillin-streptomycin (pH 7.4). Cells were incubated at 37 °C in a humidified air atmosphere of 5% CO_2_. The medium was changed every 2–3 days. Upon reaching 90% confluence, cells were washed with PBS and detached with a 0.05% trypsin-EDTA solution for 5 min (37 °C, 5% CO_2_). Trypsin was inhibited by adding culture medium and was then removed by centrifugation (300× *g*, 5 min). Cells were used up to passage 22.

Membrane samples were cut with a diameter of 6 mm and sterilized first in 70% ethanol and second by UV light exposure (30 min), after which the membranes were washed in PBS three times before being immersed in DMEM to achieve full swelling. A cell density of 2 × 10^4^ cells.cm^−2^ suspended in DMEM was seeded on each membrane sample and on TCPS (positive control) inside 48-well plates. The cultures were incubated at 37 °C in a humidified air atmosphere (5% CO_2_) for one, four, and seven days.

Cell viability was determined after each time point using an MTS colorimetric assay (Cell Titer 96 Aqueous One Solution Cell Proliferation Assay, Promega, WI, USA). The culture medium was completely removed, and the samples were rinsed with sterile PBS. FBS-free DMEM was then mixed with the MTS reagent at a 5:1 ratio and added to each well, which was followed by an incubation period of 3 h (37 °C, 5% CO_2_). The absorbance was measured in triplicate at 490 nm in a new 96-well plate using a light-protected microplate reader (Synergy HT, BioTEK, Santa Clara, CA, USA). The results for each condition were expressed through the absorbance values normalized by the DNA content as a function of culture time.

Cell proliferation was determined using the PicoGreen dsDNA kit (Thermo Fisher, Waltham, MA, USA). PicoGreen is a double-stranded DNA (dsDNA) fluorophore with a high sensitivity to low levels of dsDNA and an insensitivity to single-stranded DNA (ssDNA) and other contaminants [24]. Cell cultured specimens were first washed twice with sterile PBS and then subjected to osmotic (by adding ultrapure water) and thermal shocks (by freezing the cells at −80 °C for at least 1 h). The PicoGreen dsDNA kit was used as instructed by the supplier. The recovered supernatant was read on a microplate reader (BioTek, Santa Clara, CA, USA) using excitation and emission wavelengths of 485 and 528 nm, respectively. Each sample was measured in triplicate, and the amount of DNA was calculated from a standard curve that relates DNA concentration to fluorescence intensity.

The cell viability and cell proliferation studies were performed in triplicate for every condition.

### 2.15. Statistical Analysis

Statistical analysis was performed to determine significant differences among the studied parameters. Different tests were used depending on the number of test groups and the sample size. The statistical analysis was conducted with the software GraphPad Prism version 8.0 (GraphPad Software Inc., San Diego, CA, USA). The values were considered different for a level of significance of *p* < 0.05 (minimum of 95% confidence interval).

## 3. Results and Discussion

### 3.1. UV-Vis Analysis of Catechol-Modified Polymers

**Figure 2:**
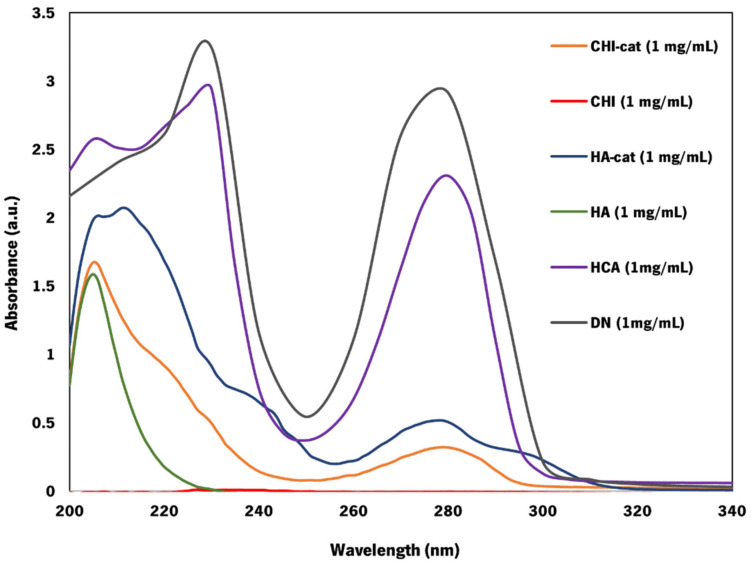
UV-vis spectra of CHI, HA, CHI–cat, HA–cat, HCA, and DN (λ = 200–320 nm). The absorbance is presented in arbitrary units. (CHI was dissolved in a 0.15 M NaCl solution (1.5% (*v*/*v*) acetic acid solution as solvent), and CHI–cat, HA, HA–cat, HCA, and DN were dissolved in a 0.15 M NaCl solution).

In order to obtain the proposed adhesive membranes, the polymeric building blocks were modified with catechol groups, resulting in the CHI–cat and HA–cat DOPA analogs reported herein. UV-vis spectroscopy confirmed that CHI and HA were successfully modified with catechol groups through EDC chemistry (Figure 2). In both cases, the results were compared with a standard curve built of each catechol group donor: HCA for CHI–cat and DN for HA–cat. Both catechol-modified polymers presented the typical absorbance peak at approximately 280 nm, corresponding to the vibration of aromatic rings of catechol groups, contrary to their unmodified counterparts, as expected [20,25].

CHI–cat yielded a substitution degree of 12% through EDC chemistry and hydrocaffeic acid. While unmodified chitosan is only soluble under acidic conditions, the conjugation of catechol onto CHI’s backbone drastically increased its solubility, allowing CHI–cat to be prepared in acidic, neutral, and basic conditions. This feature and the capability of dissolving in a 0.15 M NaCl solution confirmed that the modification was successful. As for the HA–cat conjugate, the modification through EDC chemistry and dopamine hydrochloride yielded a degree of substitution of 23% and was also soluble in 0.15 M NaCl solution. Figure S1 shows characteristic functional groups of CHI and HA present in the CoPEC membranes.

### 3.2. Zeta (ζ)-Potential Measurements

**Figure 3:**
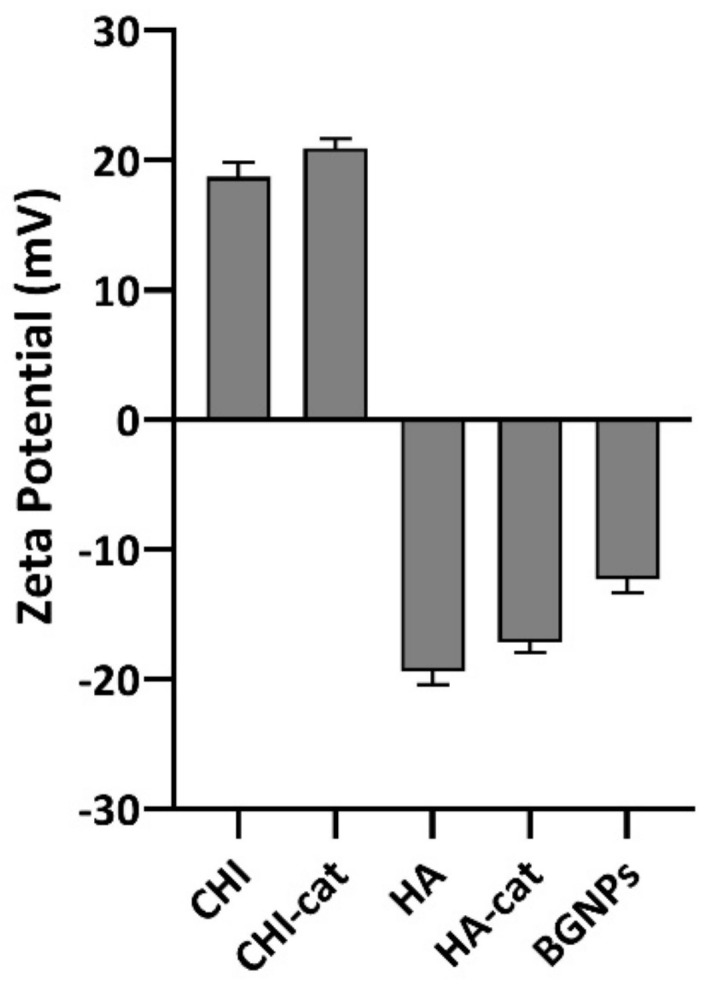
Zeta potential measurements of polyelectrolytes prepared in 0.15 M NaCl and BGNPs suspended in PBS at 37 °C.

The charge of the polyelectrolytes is the most important parameter for synthesizing polyelectrolyte complexes. Considering distinct possible compositions of CoPEC membranes, we studied the ζ-potential of the polyelectrolytes CHI/CHI–cat and HA/HA–cat as well as the BGNPs. As seen in Figure 3, both CHI and HA were confirmed as weak PEs having the expected positive and negative charges, respectively [26]. The charge of the catechol conjugates did not vary significantly from their unmodified polymers given the aforementioned low modification percentage. As for the BGNPs, they presented a ζ-potential of −12.3 ± 1.03 mV. With this value, attraction forces are stronger than the repulsion, which suggests they will have the tendency to form aggregates [27].

Additionally, we studied the mass ratios of the complexes through titrations until the ζ-potential reached ≈0 mV (Figure S2). Because the order of addition can affect the stoichiometry of the complexation [28], we observed that pouring the weakly charged polyelectrolytes in either order required similar amounts of each polyelectrolyte. However, a closer observation of the masses used revealed that pouring the negative polyelectrolyte into the positive one led to a mass ratio closer to 1:1 to achieve electroneutrality. A 1:1 ratio is characteristic of a more entropically favorable and stoichiometrically balanced complexation [29]. Thus, this order of addition was adopted in the subsequent CoPEC procedures.

### 3.3. Production of the CoPEC Membranes

We first started producing membranes via the CoPEC methodology made exclusively from polymeric materials in order to establish the feasibility of using natural polysaccharides as building blocks. Distinct combinations of materials were tested, namely, CHI/HA, CHI/HA–cat, and CHI–cat/HA–cat. These combinations correspond respectively to a control condition with the unmodified polymers, a condition with just one of the modified polymers, and a condition with both modified polymers. The control condition (CHI/HA) and the CHI/HA–cat condition always yielded membranes. However, we noticed that CHI–cat/HA–cat often coalesced as agglomerates of PECs but not as membranes. To solve this issue, we mixed equal amounts of catechol-modified polymers and unmodified materials.

The next stage consisted of incorporating BGNPs as part of the membrane composition. This was achieved by mixing them in the polycation solution. In this case, we reduced the polycation mass to accommodate a similar BGNP mass reported in a previous study [18]. The CoPEC membrane nomenclature adopted for this study is displayed in Table 1, where “P” stands for polymeric and “C” stands for composite.

### 3.4. Membranes’ Morphological and Topographic Characterization

**Figure 4:**
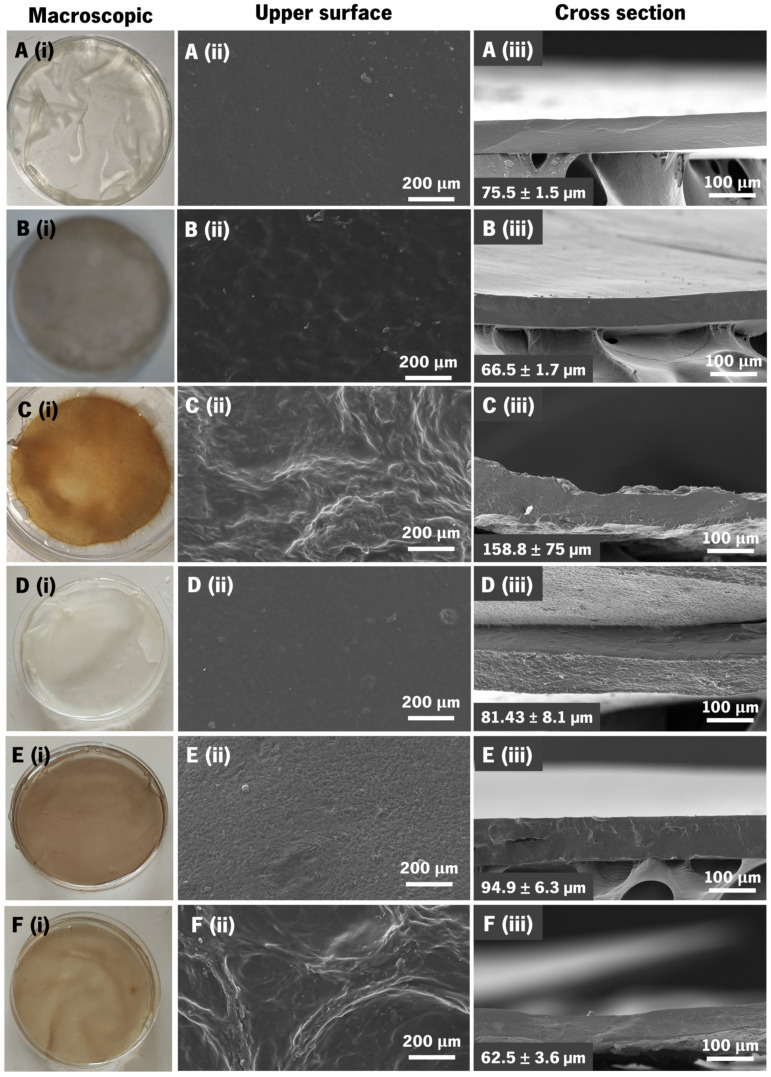
Macroscopic and microscopic views of the produced CoPEC membranes (**A**—P1; **B**—P2; **C**—P3; **D**—C1; **E**—C2; and **F**—C3): (**i**) Representative photographs in a hydrated state inside a Petri dish (diameter = 5 cm); (**ii**) SEM micrographs of membranes’ surfaces; and (**iii**) SEM micrographs of their cross-sections and respective estimated thickness in the dry state (values are means ± standard deviation).

At the macroscopic scale, the obtained membranes were homogeneous and without deformities, which facilitated easy manipulation with tweezers and even by hand (Figure 4i). All membranes were flexible and translucent in a hydrated state, with P1 being almost transparent. Only the P3 condition exhibited slight heterogeneity and a rougher texture, indicating that the higher quantity of catechol conjugate mass affects membrane topography. Although transparency is not an essential property of the membranes for the envisaged application, it can be seen that adding BGNPs did not significantly affect this aspect of the membranes, making them only slightly less transparent than the controls. Their color was mostly influenced by the use of catechol-modified polymers; the control membranes (P1 and C1) were more albescent, P2 and C2 had a greyish color due to the use of HA–cat, and C3 and P3 possessed a yellowish color due to the combination of both catechol conjugates.

Further information about the surface of the membranes was obtained by SEM. Before SEM analysis, the membranes were thoroughly washed with osmotized water to remove excess salt. The morphology of the surfaces of the CoPEC membranes and their cross-sections are shown in Figure 4. Unexpectedly, the presence of BGNPs did not significantly affect the surface roughness at this scale. Increased roughness seemed dependent on the use of both catechol conjugates, i.e., in P3 and C3 membranes. In these conditions, an increased roughness could be advantageous for cell migration and fixation to the injury site [18].

The dry thicknesses were measured from the cross-section SEM images. For each CoPEC membrane formulation, the obtained values are presented in the lower-left corner of Figure 4iii. We compared the thickness of the composite membranes with their polymeric counterparts (i.e., P1 with C1, P2 with C2, and P3 with C3). When comparing P1 to C1 and P2 to C2, the membranes containing BGNPs were the thickest, which was due to the nanoparticles’ size contribution. However, P3 was thicker than C3 despite its pure polymeric composition. We attribute this observation to the high reactivity between both catechol conjugates, which forms dense PECs that end up coalescing as thicker membranes. In C3, the relative polymeric content was lower than in P3 and smaller PECs were formed: this led to the formation of thinner membranes.

### 3.5. Water Contact Angle Measurements

**Figure 5:**
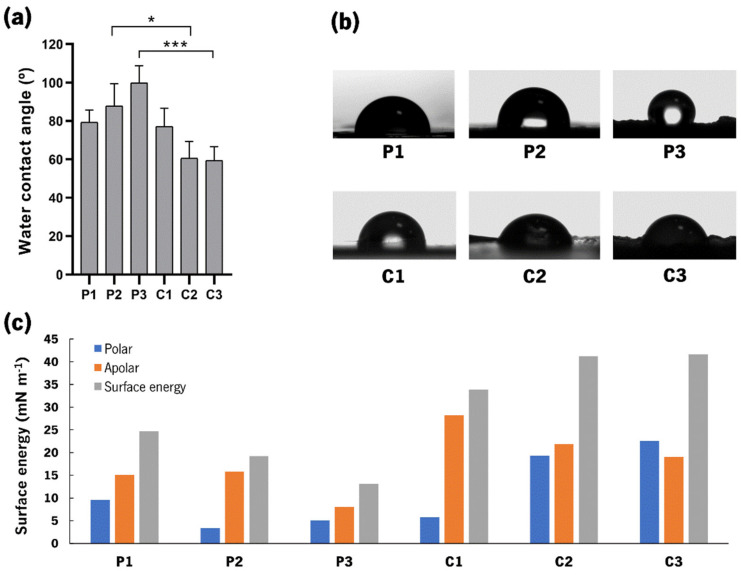
Water contact angle (WCA) measurements: (**a**) Mean and standard deviation of WCA results. Significant differences were found for *p* < 0.05 (*) and *p* < 0.001 (***); (**b**) Representative photographs of water drop shape on the surface for each membrane condition; and (**c**) Mean value of the surface energy and its apolar and polar components estimated by the OWRK method for each CoPEC membrane.

The measurement of contact angles is a fast and convenient way to distinguish between different compositions by variations in wettability. Figure 5a portrays the WCA measurements of the produced CoPEC membranes.

First, the analysis of the polymeric membranes showed a hydrophobic behavior with angles varying between ≈80 and 100°, which could be a consequence of the combination of both catechol-modified polymers. Typically, hydrophobicity is a characteristic of rougher substrates [30]; thus, the higher WCA found for P3 could be attributed to the high roughness observed in the SEM images. Second, we observed that BGNPs increased the wettability of the membranes since the average WCAs decreased with respect to their polymeric controls. These findings are in accordance with previous work [18,31,32]. Moreover, this hydrophilic behavior of membranes containing BGNPs is aligned with previous studies that describe the formation of hydration layers around hydroxyapatite materials.

Additionally, one would expect that the WCA would increase from C1 to C3 since the SEM images also showed an increase in roughness. However, the WCA decreased from ≈80° to ≈60°, respectively. This effect can be explained by the Wenzel model, which predicts that increasing the roughness of a substrate with hydrophilic components can actually amplify its wettability [33]. Because the addition of BGNPs increased the wettability of the membranes compared with the polymeric controls, the higher roughness of C3 made it the most hydrophilic CoPEC membrane of this study.

Surface energy (SE) values were also measured (Figure 5c), and the SE of the composite membranes was generally higher than their polymeric counterparts. The polar component was the major contributor, particularly for C2 and C3. Their higher SE might suggest that they could promote suitable cellular attachment. In particular, the polar component was higher than 15 mN.m^−1^, which has been associated with marked cell spreading behaviors [34].

### 3.6. Swelling and Degradation Studies

**Figure 6:**
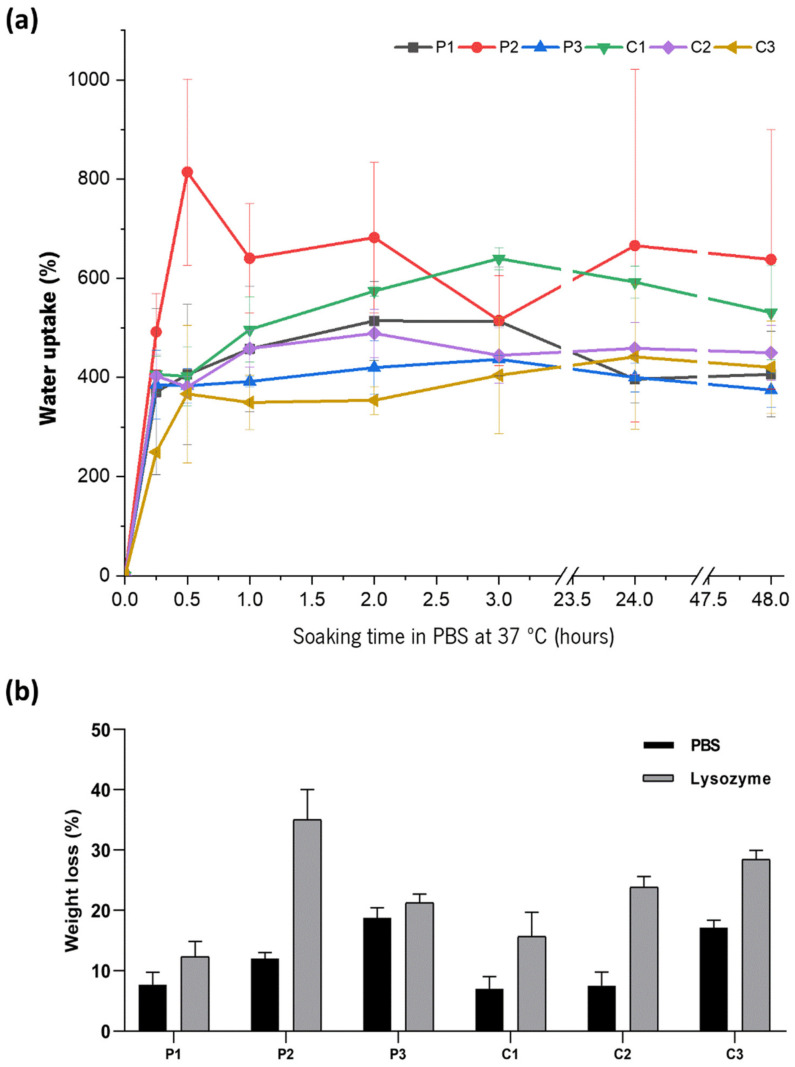
Swelling and degradation studies of the CoPEC membranes. (**a**) Water uptake of the CoPEC membranes in PBS solution for 48 h at 37 °C; (**b**) Weight loss of the CoPEC membranes after soaking in PBS and in a PBS–lysozyme enzymatic solution for 31 days at 37 °C.

Swelling of the CoPEC membranes was observed for 48 h under conditions that mimic the temperature and isotonic environment of biological media (i.e., a PBS solution with pH 7.4 at 37 °C) (Figure 6a). In the first 15 min, water uptake increased rapidly in all conditions. After 30 min, it continued to rise at a much slower rate, except for P2, where the water uptake nearly doubled in percentage. After 3 h, the general tendency for this parameter was to reach an equilibrium plateau, although P2 and C1 only stabilized between 24 h and 48 h. A comparison between the composite conditions and their polymeric controls indicated that the presence of the BGNPs increased *WU*. A possible explanation lies in the fact that BGNPs increase the hydrophilicity of the membranes, as indicated by the aforementioned WCA measurements. The exception to this hypothesis was the high *WU* exhibited by P2. Additionally, the lower *WU* values obtained from membranes incorporating catechol moieties was expected, as described in previously works [35,36,37]. This indicates that HA–cat could present a higher affinity with water than the other CoPEC building blocks (CHI/CHI–cat), leading to an increased amount of absorbed water by the membrane.

Several enzymes may oxidize or hydrolyze biopolymers used in biomedical devices when they come into contact with bodily fluids and tissues. These reactions affect host responses, cell proliferation, and ultimately regeneration of native tissue [38,39]. For GTR membranes, structural and mechanical integrity is expected for four to six weeks [40,41]. We evaluated the enzyme-catalyzed hydrolysis of the CoPEC membranes in a lysozyme solution, as this enzyme cleaves the 1,4-beta-linkages between N-acetyl-D-glucosamine residues of chitosan [42]. Figure 6b shows the weight loss of the produced membranes after 31 days. As anticipated, all the CoPEC membranes showed higher *WL* values while immersed in the enzymatic solution than the controls in the PBS solution.

Next, we analyzed the effect of the BGNPs and catechol groups on the enzymatic degradation. First, regarding the BGNPs, there was a tendency for the *WL* of composite membranes to increase when compared with their pure polymeric counterparts. This was due to the release of the BGNPs from the degrading polymeric matrix, which amounts to the detachment of more mass, in agreement with previous studies [18,31]. Second, we observed a tendency for the *WL* to increase with the number of catechol conjugates, indicating to some extent that degradation is proportional to the catechol group content. The exception to this logic was P2, as it presented the highest *WL* of all conditions. This could be correlated to the water uptake of P2, which was the highest measured. In fact, a faster degradation rate was likely enabled by the swelling of this membrane [43,44], which facilitates the action of the enzyme. The dissolution of the BGNPs from the degrading polymeric matrix may be relevant for the bioactivity of the membranes, which will be investigated later.

### 3.7. Mechanical Properties

**Figure 7:**
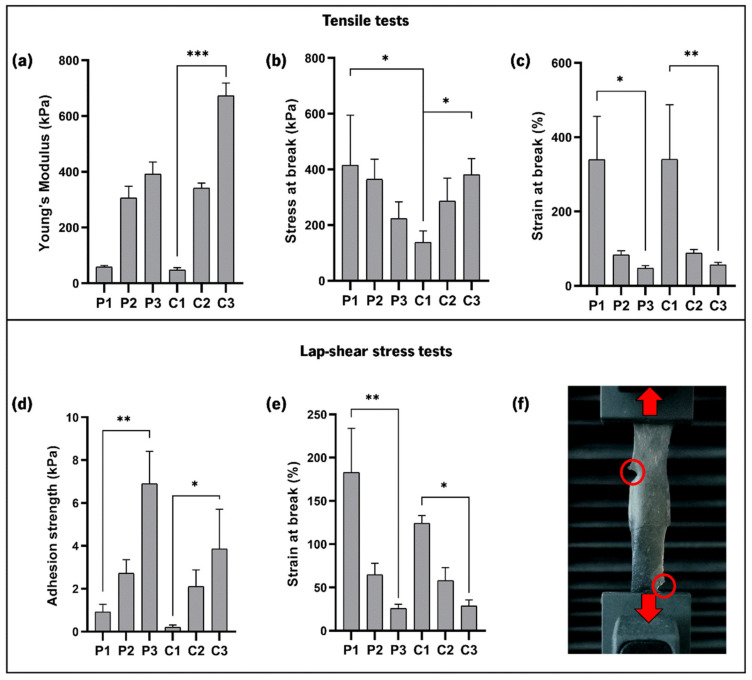
Tensile and lap-shear stress tests of the CoPEC membranes and the various measured parameters. (**a**) Young’s modulus; (**b**) Stress at break; (**c**) Strain at break; (**d**) Adhesion strength; (**e**) Strain at break (lap-shear); (**f**) Representative picture of a lap-shear stress test with a CoPEC membrane showing self-healing and the rupture points highlighted in red circles. Significant differences were found for *p* < 0.05 (*), *p* < 0.01 (**), and *p* < 0.001 (***).

The stiffness of the CoPEC membranes was measured to determine their suitability for GBR. Indeed, improper mechanical properties may elicit aberrant mechanotransduction in cells, which is inadequate for the desired application [45]. The Young’s moduli of P1 and C1 were lower (0.06 ± 0.01 MPa and 0.05 ± 0.01 MPa, respectively) than the remaining conditions, and P3 and C3 were the stiffest membranes (0.39 ± 0.04 MPa and 0.68 ± 0.04 MPa, respectively) (Figure 7a). The tensile strain was drastically lower for membranes containing catechol-modified polymers, especially in P3 and C3, where both CHI–cat and HA–cat were used (Figure 7c). C3 was undoubtedly the stiffest and the least plastic (i.e., it presented the lowest deformation), making it the most reliable condition to deal with mechanical loads. We attribute C3’s performance to its catechol conjugate content paired with the inclusion of the bioceramic nanoparticles. Compared with previous studies [15,46], the maximum Young’s modulus values presented herein are lower. Still, the membranes were less brittle and more flexible since the strain values range between 340 and 47%. Based on this mechanical performance, the proposed CoPEC membranes could be alternative biodegradable membranes suitable to support the interfacial regeneration of soft and hard tissue.

Lap-shear stress tests were conducted to quantify the adhesiveness of the CoPEC membranes. When preparing the tested specimens, all membranes showed some extent of self-healing ability, as they fused after overlapping (Figure 7f). Moreover, none of the membranes ruptured at the overlapping area. This is novel with respect to LbL membranes previously produced by our group, in which the dipping methodology and the same polysaccharides and BG nanoparticles were used. In those films, the joined segments slip at the interface of contact under stress [15]. The fusion of overlapped CoPEC constructs P1 and C1 (i.e., without catechol group content) could be related to the high capacity of HA to absorb high amounts of water, which grants high molecular mobility to the surfaces and an ability to establish new bonds with each other [47]. In the remaining membranes, fusion may be amplified by the intrinsic mucoadhesive properties of both catechol-modified polymers.

Figure 7d shows, as expected, that the adhesiveness of the CoPEC membranes increased with the combination of both catechol-modified polymers, with P3 and C3 presenting the highest lap-shear stress at break (6.90 ± 1.50 kPa and 3.85 ± 1.85 kPa, respectively). All composite membranes showed lower adhesion strength than their polymeric controls, which is in accordance with previous findings reported for LbL free-standing membranes [15]. This is likely due to the nanoparticles adding small topographic features to the overlapping area, causing incomplete contact between the surfaces. This behavior is akin to solid/viscoelastic and elastic contacts, where the real contact area is lower than the apparent contact area [48,49,50]. Additionally, it was determined that using catechol-modified polymers successively lowered the strain of the overlapped area at break. Therefore, the P3 and C3 conditions were the most adhesive and the most resistant to deformation under stress (25.6 ± 5 % and 28.7 ± 6.7 %, respectively). Given that commercial bioadhesives such as fibrin, chitosan gel, and cyanoacrylate-based materials have a shear adhesive strength between 4 and 68 kPa [51], it can be affirmed that the proposed CoPEC membranes have promising adhesive properties since they are within the range of values exhibited by the above-mentioned well-known tissue glues. Such enhanced adhesion could certainly pose an alternative to the existing materials in helping the implantation of membranes in situ.

**Figure 8:**
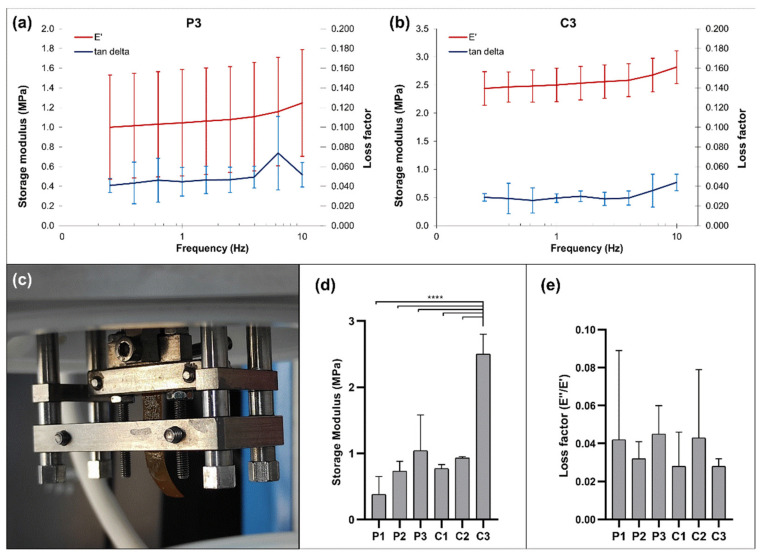
DMA measurements of the CoPEC membranes: Variation of the storage modulus and loss factor along a frequency scan ranging from 0.1 to 10 Hz in PBS solution at 37 °C for P3 (**a**) and C3 (**b**); (**c**) Experimental setup; mean values obtained at physiological frequency (1 Hz) for: (**d**) Storage modulus and (**e**) loss factor. Significant differences were found for *p* < 0.0001 (****).

The mechanical and viscoelastic properties of the CoPEC membranes were evaluated by dynamic mechanical analysis (DMA). The storage modulus (E′) and the loss factor (E′′/E′) as a function of the tensile loading frequency for the P3 and C3 conditions are presented in Figure 8a,b. Special attention was paid to these compositions since their tensile stress and lap-shear stress outperformed the other membrane conditions in almost every aspect. The experimental DMA results obtained for the remaining conditions are shown in Figure S3.

The results display an increase in E’ with the frequency increase, as is typical from viscoelastic materials [52]. This increase was most prominent with C3, implying that the combined effect of the catechol-modified polymers and the BGNPs led to superior membrane stiffness. We then looked specifically at the values obtained at a frequency of 1 Hz, which is considered to be the frequency of the standing human body in unidirectional motion [53] (Figure 8d,e). The composite membranes tended to exhibit better dynamic mechanical performance than their respective pure polymeric formulation. This was more evident for C3 since its storage modulus was significantly higher than the other compositions. These findings are consistent with the results of the static mechanical characterization, where C3 already displayed the most promising mechanical performance.

### 3.8. In Vitro Mineralization Studies

**Figure 9:**
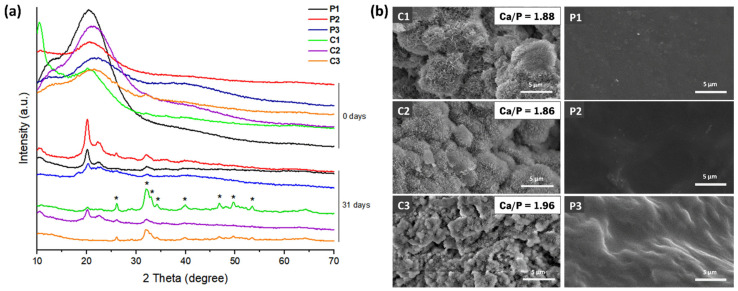
In vitro mineralization study: (**a**) XRD spectra of the CoPEC membranes before and after immersion in SBF for 31 days (reference hydroxyapatite peaks are indicated as *); (**b**) SEM micrographs of CoPEC membranes’ surface after immersion in SBF for 31 days. Scale bars represent 5 µm.

The ability of the CoPEC membranes to promote the formation of a hydroxyapatite layer on their surfaces was evaluated in vitro after their immersion in SBF. XRD spectra of all membrane conditions before SBF immersion (i.e., zero days) fundamentally confirmed the amorphous profile of the analyzed surfaces from 2θ =15° to 35°, as calcium phosphate had not promoted the growth of a crystalline phase yet (Figure 9a). After 31 days, major diffraction peaks associated with apatite appeared, namely, 2θ = 26° and 2θ = 35°. Other apatite-related peaks with lower intensity could also be identified at 2θ = 32.9, 34.7°, 39.8°, 46.7°, 49.7°, and 53.1°. The XRD spectra had the typical hydroxyapatite diffractogram patterns [54], confirming the bioactive behavior of the BGNP-containing membranes.

SEM and EDS were further conducted to provide a better understanding of the nature of the formed calcium phosphate layer. The SEM surface images showed nucleation and growth of apatite crystals for all composite formulations (Figure 9b). As expected, only the formulations containing BGNPs induced the formation of crystals that resembled apatite-like structures on their surfaces. The precipitate displayed a distinctive cauliflower morphology, containing needle-like nanometric structures typical of bone-like apatite [23]. The EDS analysis confirmed the elemental contents of silicon (Si), phosphorus (P), and calcium (Ca) on the membrane’s surface. Moreover, the obtained Ca/P ratios were close to 1.9, which is higher than the stoichiometric ratio of 1.67 [55]. Constructs with values above the stoichiometric ratio have been shown to improve cellular adhesion [56]. Therefore, we did not anticipate any deleterious response from osteoblast cells in contact with these membranes.

### 3.9. Cellular Assays

**Figure 10:**
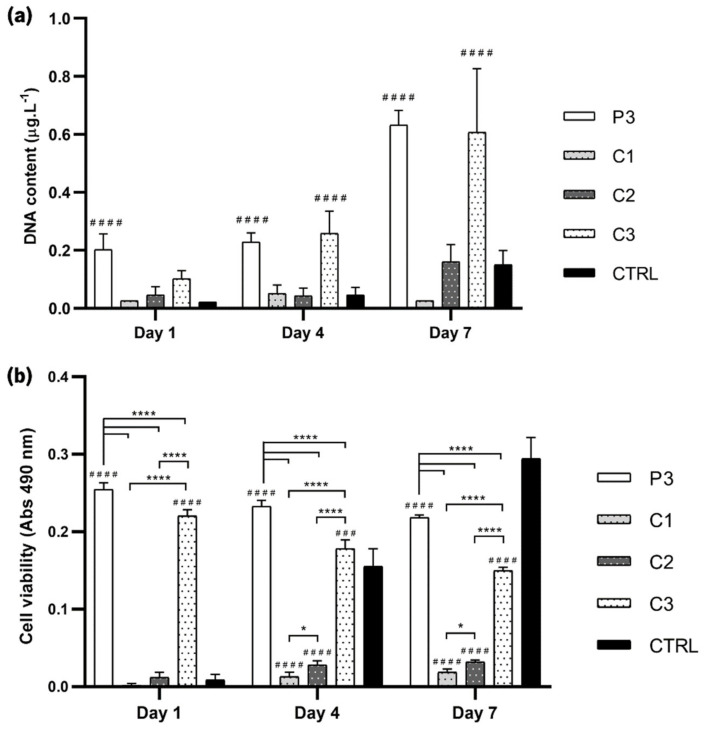
Cellular assays. (**a**) DNA quantification using the PicoGreen assay; (**b**) Cellular viability analysis using the MTS assay. SaOs-2 cells cultured on the P3, C1, C2, and C3 membranes in D-MEM for one, three, and seven days. TCPS was used as a control. Significant differences were found for *p* < 0.05 (*) and *p* < 0.0001 (****). Significant differences between membrane conditions with the control (CTRL) are represented as (#).

For proof of concept for envisaging GBR, the response of SaOs-2 osteoblast-like cells on the various CoPEC membranes was studied. Cellular viability and proliferation were measured by direct contact of SaOs-2 cells at one, four, and seven days with selected CoPEC compositions. Based on the favorable mechanical performance of P3 and the bioactive behavior of C1, C2, and C3, these conditions were selected for the cellular assays. First, cell proliferation was assessed by DNA quantification (Figure 10a). In general, the CoPEC membranes proved to be successful in promoting cell proliferation. This was most clearly observed in P3 and C3 membranes. The roughness of these substrates was likely the determining factor for good osteoblast adhesion since, as previously mentioned, cells usually prefer rough substrates. It is also possible that the combination of both catechol-functionalized polymers contributed to cell adhesion to some extent since hydroxyl groups—present in catechol groups—have been implicated in enhanced cell and tissue adhesion [57]. Notably, osteoblasts did not adhere on C2 as much as on P3 and C3 despite showing a polar component of the surface energy of ≈20 mN.m^−1^ (i.e., >15 mN.m^−1^). In fact, the adhesion was greater on P3 even though its polar component was just ≈5 mN.m^−1^. This is a strong indicator that the surface energy was not a major determinant in driving cell adhesion. Figure S4 complements the DNA assay by estimating the number of SaOs-2 cells in the CoPEC membranes.

Cellular viability was then evaluated by the MTS assay, where the metabolic activity was measured through the chemical reduction of the MTS compound to formazan. The cell viability increased over time when seeded on C1 and C2, but cells on P3 and C3 showed far better viability. Notably, in these two conditions, the cell viability was the highest in day one but was slightly decreased afterward (Figure 10b). Thus, the data indicate that using two catechol-based polymers in the CoPEC composition instead of one resulted in a slight cytotoxic impact over time despite the significantly high viability. This behavior was unexpected since other coatings made with similar dopamine-modified HA compositions are non-cytotoxic [15,18]. We hypothesize that using two catechol-modified species increases the probability of redox chemistry reactions, which are known to induce cytotoxicity on both microorganisms and mammalian cells [58]. Nonetheless, we highlight that the impact on viability was minor, as the absorbance decreased less than 20% between day one and day seven.

These cellular assays revealed that CoPEC membranes using both catechol-functionalized HA and CHI greatly enhanced the cell proliferation and viability of these membranes, making them mechanically and biologically suitable adhesives. The fact that CHI–cat/HA–cat membranes doped with BGNPs also elicited a strong positive effect on the activity of osteoblast-like cells makes them adhesives with the added capacity for hydroxyapatite mineralization, which is important for the regeneration of bone.

## 4. Conclusions

This work demonstrated the successful assembly of six different formulations of membranes using the CoPEC methodology based on CHI, CHI–cat, HA, HA–cat, and BGNPs. This alternative to LbL proved to be a straightforward method, where the complexation of the building blocks involves only one step, followed by a coalescence period, without cross-linking procedures. An extensive and multi-faceted characterization was performed, which revealed the benefits of using both catechol conjugates simultaneously. The overall ideal condition was the one that contained a mixture of 15% BGNPs and all modified and non-modified polymers (i.e., 17.5% CHI, 17.5% CHI–cat, 25% HA, and 25% HA–cat). Specifically, membranes with this formulation lost as little as 30% of their weight in 31 days. The inclusion of BGNPs also improved the Young’s modulus about two-fold compared with purely polymeric membranes, making them more suitable for the regeneration of hard tissues. The observed hydroxyapatite formation on composite membranes presented a Ca/P ratio of about 1.9, which further increases the suitability for GBR. This became evident by observing the positive impact elicited on the activity of osteoblasts. The successful production and incorporation of the BGNPs on the CoPEC membranes tried to reproduce the inorganic content reported in previous works. This is a landmark since, to our knowledge, organic/inorganic membranes had yet to be achieved through this technique. Furthermore, the ceramic component not only granted mineralization potential but also improved mechanical properties while also influencing other characteristics, such as wettability, swelling, and adhesiveness. The use of BGNPs is aligned with the current trends of supplementing orthopedic devices with bioceramics to mimic the mineral phase found in bone. In fact, this composition possessed bioactive capabilities, enhanced mechanical properties, and increased cellular behavior that makes such membranes potentially suitable for GBR.

## Figures and Tables

**Table 1:** CoPEC membrane compositions and percentage of each material in relation to the total membrane mass.

Formulation	Polycation	Polyanion	Ceramic
**P1**	CHI (50 %)	HA (50 %)	-
**P2**	CHI (50 %)	HA–cat (50 %)	-
**P3**	CHI (25 %) and CHI–cat (25 %)	HA (25 %) and HA–cat (25 %)	-
**C1**	CHI (35 %)	HA (50 %)	BGNPs (15 %)
**C2**	CHI (35 %)	HA–cat (50 %)	BGNPs (15 %)
**C3**	CHI (17.5 %) and CHI–cat (17.5 %)	HA (25 %) and HA–cat (25 %)	BGNPs (15 %)

## Data Availability

The data presented in this study are available on request from the corresponding author.

## Supplementary Materials

The following supporting information can be downloaded at: https://www.mdpi.com/article/10.3390/jfb14010003/s1, Figures S1–S4 (References [59,60,61,62,63,64,65] are cited in the Supplementary Materials).
