# Supplementary material for: Exploiting Polyelectrolyte Complexation for the Development of Adhesive and Bioactive Membranes Envisaging Guided Tissue Regeneration
Source: J Funct Biomater. 2022 Dec 20;14(1):3. doi: 10.3390/jfb14010003 (PMC9864365; doi:10.3390/jfb14010003)
Supplement: Supplementary file 1 [file jfb-14-00003-s001.zip › jfb-2029369-supplementary.pdf]

Supplementary Materials

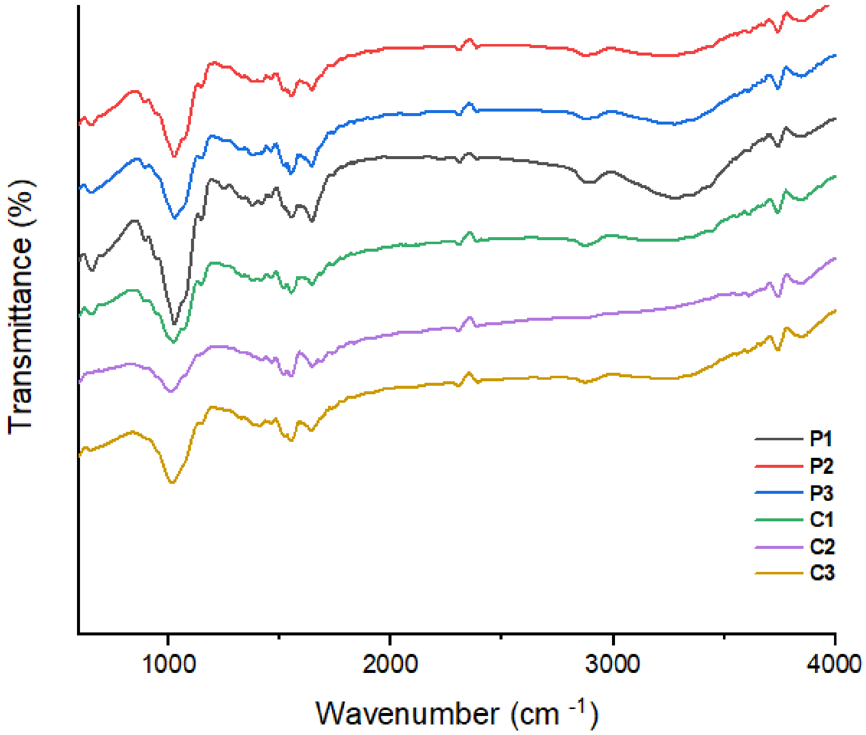

Figure S1 - FTIR spectra of distinct CoPEC membranes.

The FT-IR results of distinct CoPEC membranes are shown in Figure S1. All membranes exhibit the characteristic bands of CHI: at 3267 cm^{-1} from the amine and hydroxyl groups [59]; at 1392 and 1565 cm^{-1} from the amide II of group (O=C-N-H) [60]; at 926 cm^{-1} to 1151 cm^{-1} there are the vibrational bands of CHI [59,61-63] and bands at 1281,1026 and 1078 cm^{-1} are attributed to the presence of OH-, C-O and C-N stretching [1], respectively; at 1620 and 1660 cm^{-1} and between 1630 and 1700 cm^{-1} correspond to C=O bands and amide I [61,63], respectively.

The characteristic peaks of HA are also detected: at 3383 cm^{-1}, which corresponds to the –OH– [64], also detectable in a range of 3350 to 3250 cm^{-1}; the methylene group appears around 2940 and 2920 cm^{-1} and amide bonds stretching (C=O) are detectable in a range of 1700 to 1250 cm^{-1}; the peak at 1560 cm^{-1} corresponds to N-H groups; and the peak at 1100 cm^{-1} corresponds to ethers groups (-C-O-C) [65]; the C-O and C-O-C stretching modes are detected at 1132 cm^{-1} [61].

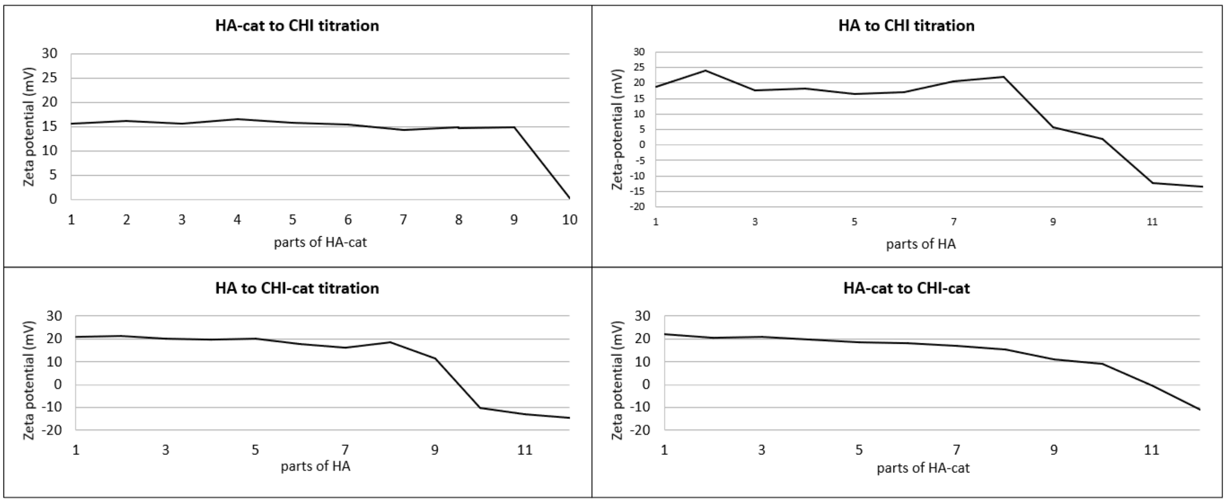

Figure S2 - Zeta potential titration studies with natural polymers (CHI, HA) and their respective catechol-conjugates (CHI-cat, HA-cat).

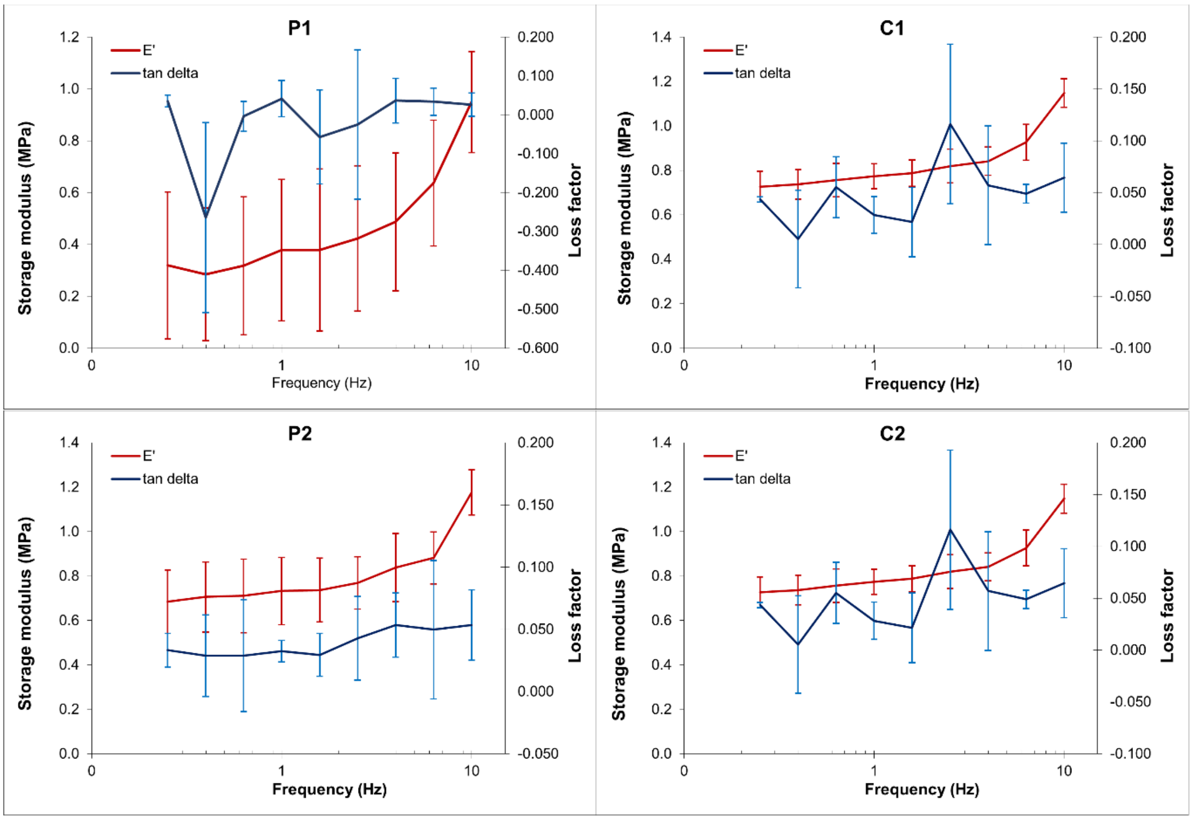

Figure S3 – DMA spectra with the storage modulus (E') and the loss factor (E''/E') as a function of the frequency obtained for CoPEC membranes P1, P2, C1 and C2 hydrated in PBS solution at 37ºC.

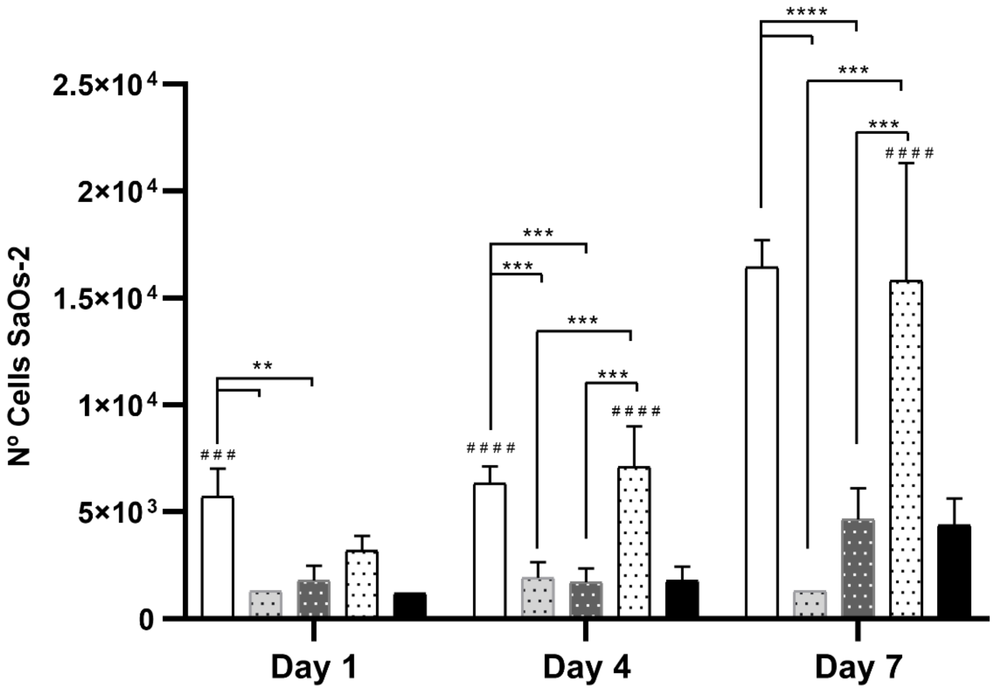

Figure S4 - DNA assay: Estimated number of SaOs-2 cells after 1, 4 and 7 days. Significant differences were found for $p < 0.01$ (**), $p < 0.001$ (***), and $p < 0.0001$ (****). Significant differences between any condition with the control (CTRL) are represented as (#).
